# Effects of Uncertainty on Depression in Women Undergoing Assisted Reproductive Technology: The Mediating Role of Perceived Stigma and the Moderated Mediation by Spousal Support

**DOI:** 10.1111/nhs.70305

**Published:** 2026-02-18

**Authors:** Miok Kim

**Affiliations:** ^1^ Department of Nursing, College of Nursing Dankook University Cheonan South Korea

**Keywords:** assisted reproductive technology, depression, infertility, social support, stigma, uncertainty

## Abstract

Women undergoing assisted reproductive technology (ART) often experience depression linked to treatment‐related uncertainty. This study examined whether perceived stigma mediates the relationship between uncertainty and depression and whether spousal support moderates this effect. PROCESS Macro Model 14 was used, controlling for miscarriage experience, counseling history, and spousal proactiveness. Conditional indirect effects and the moderated mediation index were assessed using bootstrapping with 95% confidence intervals. Uncertainty significantly predicted perceived stigma (*β* = 0.56, *p* < 0.001) and depression (*β* = 0.54, *p* = 0.002). Perceived stigma also significantly affected depression (*β* = 0.61, *p* < 0.001). However, spousal support (*β* = −0.09, *p* = 0.617) and its interaction with stigma (*β* = 0.02, *p* = 0.913) were not significant. While indirect effects remained significant at all spousal support levels, the moderated mediation index was nonsignificant (95% CI: −0.15 to 0.21). Perceived stigma mediates the effect of uncertainty on depression, but spousal support does not moderate this pathway. Interventions should target uncertainty and stigma reduction to improve mental health in women undergoing ART.

## Key Points

- Uncertainty about infertility treatment significantly increases depression in women undergoing ART.
- Perceived stigma mediates the relationship between uncertainty and depression, whereas spousal support does not significantly moderate this mediation.
- To reduce psychological distress, interventions should focus on minimizing treatment‐related uncertainty and reducing internalized stigma through identity‐affirming and coping‐oriented strategies.

## 1 Introduction

Infertility is one of the major issues in reproductive health. The World Health Organization (WHO) emphasizes that neglecting infertility can lead to widespread psychological problems at both individual and societal levels, highlighting the need for greater attention and active support for infertility‐related issues (García‐Blanco et al. 2018). Women undergoing assisted reproductive technology (ART) experience not only the stress of infertility itself but also anxiety and depression due to prolonged treatment and uncertainty about outcomes (Maroufizadeh et al. 2019). In general, women are more likely than men to experience psychological disorders and reduced quality of life (Maroufizadeh et al. 2018). In Korea, more than 50% of women undergoing infertility treatment report emotional changes such as anxiety, anger/irritability, depression, helplessness, despair, and sleep disturbances during ART procedures, and approximately 25% have had thoughts of suicide (Kim et al. 2021).

In infertility treatment, the passage of time is considered a critical factor due to the decline in fertility associated with aging‐related biological causes (Mitrović et al. 2022). Since the success rate of a single in vitro fertilization (IVF) cycle is only about 25%, most couples experience repeated treatments and failures (Aimagambetova et al. 2020). Uncertainty about how long infertility treatment will take is closely related to depression in women undergoing such treatments. This depression is associated with the possibility of treatment failure, financial burden, social pressure, concerns about treatment procedures and technologies, and fears about the continuation of fertility (Taskin et al. 2016). Previous experiences of treatment failure further increase uncertainty regarding whether to continue treatment and the likelihood of success (Rooney and Domar 2016). These psychological burdens can also negatively affect hormonal, neuroendocrine, or immune functions, which may adversely impact ART outcomes (Aimagambetova et al. 2020). Therefore, how women perceive, interpret, and respond to the uncertainty surrounding infertility and its treatment significantly affects the severity and duration of psychological difficulties such as depression (Gentes and Ruscio 2011).

Stigma, defined by a sense of suppressed identity and inadequacy, is closely associated with the social and emotional aspects of infertility. In the context of infertility, stigma often manifests as self‐stigma, whereby individuals experiencing infertility may develop negative self‐perceptions and feelings of social isolation when they perceive themselves as different from others based on their social experiences (Younesi et al. 2006). The perception of stigma can lead to a sense of identity threat, and when coping strategies are ineffective, this can result in depression (Major and O'Brien 2005). This occurs because individuals who identify with or belong to a stigmatized group tend to internalize the public's negative perception of that group (Corrigan et al. 2009), leading to feelings of shame and experiences of discrimination when they feel unable to meet societal expectations (Davern and O'Donnell 2018). Moreover, stigma is reflected in empirical evidence showing that over 50% of Korean women experiencing infertility have reported facing prejudice due to their condition (Jeong and Kang 2017). In this context, women with infertility may also experience perceived public stigma, whereby they believe they are viewed negatively by members of society (Kim and Ban 2022). Therefore, it is important to examine how the level of perceived stigma among women undergoing assisted reproductive technology influences the relationship between uncertainty and depression.

For women, reproductive challenges can affect their sense of identity, which may lead to identity‐related uncertainty. This uncertainty can in turn increase perceived difficulties in communication and relationship uncertainty with their spouse (Yoon and Theiss 2021). Over time, women who are unable to conceive may experience anxiety or fear of separation from their partner (Kucukkelepce and Unver 2022), which, combined with the unpredictable nature of infertility treatment, becomes a major source of depression (Mandrik et al. 2015). Spousal support plays a protective role against psychological and emotional distress, such as depression, during infertility treatment (Dadhwal et al. 2022), and is one of the key factors affecting fertility adjustment (Kucukkelepce and Unver 2022). Although higher levels of social support have been shown to alleviate infertility‐related depression (Ozturk et al. 2021), many women refrain from sharing their struggles with family or friends, thus failing to receive the support they need (Tabyshalieva 1997). On the other hand, excessive involvement or unrealistic expectations from family can increase psychological burdens, and peer support may not effectively predict or alleviate infertility‐related distress (Patel et al. 2018). This highlights the central role of spousal support and suggests the need to explore the mechanisms through which it impacts psychological distress.

When there is a correlation between an independent and dependent variable, and the mechanism underlying this relationship is being examined, mediation analysis is used. When the effect of the mediator on the dependent variable depends on a certain condition or context, moderated mediation analysis is employed (Hayes 2022). Moderated mediation implies that the strength of the mediating effect is influenced by the level of a moderator variable (Ryu et al. 2022). Infertility is one of the most significant stressors causing psychological distress (Maroufizadeh et al. 2019). Among women undergoing infertility treatment, high levels of illness uncertainty and perceived stigma have been associated with increased psychological distress (Davis et al. 2010). Perceived support from family and the social environment affects both perceived stigma and depression (Ozturk et al. 2021), while spousal support serves as a protective factor against depression in women with infertility (Dadhwal et al. 2022). Uncertainty is significantly related to elevated levels of depression in these women (Kamisli et al. 2021), and contributes to reduced quality of life (Lee et al. 2020).

Based on these previous findings, it can be hypothesized that the mediating effect of perceived stigma in the relationship between uncertainty and depression may vary depending on the level of spousal support. Although prior studies have tested causal relationships and simple mediation models, few have empirically examined the moderated mediation effect‐how spousal support influences the conditions under which depression occurs. Therefore, this study aims to investigate the relationship between uncertainty and depression among women undergoing ART, with a specific focus on how perceived stigma mediates this relationship depending on the level of spousal support. The findings are expected to provide foundational data for developing effective nursing interventions to alleviate the psychological and emotional distress of women facing infertility.

## 2 Conceptual Framework and Study Objectives

**FIGURE 1:**
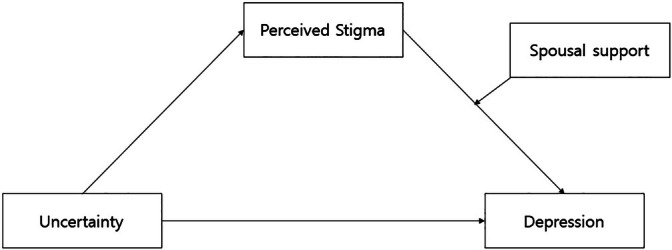
Mediation effect model of perceived stigma and moderated mediation effect model of spousal support.

The purpose of this study is to establish and test a conceptual model that explains the mediating effect of perceived stigma and the moderated mediation effect of spousal support on the relationship between uncertainty and depression among women undergoing infertility treatment. Previous studies have shown that uncertainty experienced by women undergoing infertility treatment influences both perceived stigma (Kamisli et al. 2021) and depression (Taskin et al. 2016; Kamisli et al. 2021), and that perceived stigma itself is associated with increased levels of depression (Rooney and Domar 2016). Social support plays a significant role in reducing perceived stigma and alleviating depression (Ezzati et al. 2013), while the level of spousal support perceived by women acts as a protective factor against depression (Dadhwal et al. 2022). Based on these findings, this study proposes a conceptual model in which perceived stigma mediates the relationship between uncertainty and depression, and spousal support moderates this mediating effect (Figure 1).

The specific objectives of this study, based on the conceptual model, are as follows:

To identify differences in uncertainty, depression, perceived stigma, and spousal support according to participants' characteristics.

To examine the relationships among uncertainty, depression, perceived stigma, and spousal support.

To investigate the mediating effect of perceived stigma on the relationship between uncertainty and depression.

To test the moderated mediation effect of spousal support on the relationship between uncertainty and depression through perceived stigma.

## 3 Methods

### 3.1 Research Design

This cross‐sectional study aimed to examine the mediating effect of perceived stigma and the moderated mediation effect of spousal support on the relationship between uncertainty and depression in women undergoing infertility treatment. This study was conducted and reported in accordance with the STROBE (Strengthening the Reporting of Observational Studies in Epidemiology) guidelines for cross‐sectional studies.

### 3.2 Participants

This study employed convenience sampling to recruit women undergoing ART at a specialized infertility clinic (located in Busan, South Korea). Previous studies have shown that psychological distress related to infertility is more pronounced in women without children compared to those with children (Donkor and Sandall 2007), and is heightened among those who have experienced repeated treatment failures (Patel et al. 2018; Rahimi et al. 2021). Based on this evidence, inclusion criteria were established as follows:

- Married women aged 20–44 years without children
- Women who had been diagnosed with infertility (due to female, male, combined, or unexplained factors) had experienced at least one failed ART cycle and were currently undergoing ART
- Women who were able to complete a self‐report questionnaire and had provided informed consent after understanding the study's purpose.

Exclusion criteria included:

- Women with language or cognitive impairments that would hinder understanding and completing the questionnaire
- Those with other physical or mental illnesses besides infertility, and
- Women undergoing ART solely for fertility preservation purposes.

## 4 Data Collection

Data collection was conducted from January 3 to June 1, 2022, following approval from the participating medical institution. Recruitment posters were displayed and distributed within the hospital. Women interested in participating were approached by trained research assistants, who explained the study's purpose and procedures. Written informed consent was obtained before distributing the survey.

Sample size was calculated using G*Power 3.1.9.4 software based on multiple regression analysis. With a significance level (*α*) of 0.05, a statistical power (1‐*β*) of 80%, and a medium effect size of 0.15, the minimum required sample size was 139 participants, assuming 15 predictor variables (including demographic and study variables). Considering a 10% dropout rate, 150 questionnaires were distributed. Of these, 131 were returned (response rate: 87.3%). After excluding 13 questionnaires due to multiple responses or missing data, a total of 118 were included in the final analysis.

## 5 Instruments

The instruments used in this study measured uncertainty, depression, perceived stigma, and spousal support. Permission was obtained for tools that required authorization from the original developers or translators. All instruments used in this study demonstrated good to excellent internal consistency, supporting their reliability for the present analysis.

### 5.1 Uncertainty

Participants' uncertainty was measured using the infertility uncertainty scale developed by Kim and Kim (2010). This tool consists of 10 items: 4 items assessing relational aspects and 6 items assessing personal aspects of uncertainty experienced by women with infertility. Each item is rated on a 5‐point Likert scale ranging from 1 (“Very certain”) to 5 (“Not certain at all”), with higher scores indicating greater uncertainty about infertility. The reliability of the tool was Cronbach's *α* = 0.78 at the time of development, and 0.83 in this study.

### 5.2 Depression

Depression was measured using the Depression subscale (DASS‐D) of the Depression Anxiety Stress Scales (DASS) developed by Lovibond and Lovibond (Lovibond and Lovibond 1995) and validated in Korean by Jun et al. (Jun et al. 2018). The scale includes 7 items that reflect low positive affect, low self‐esteem and motivation, and hopelessness. Each item is rated on a 4‐point Likert scale from 0 (“Did not apply to me at all”) to 3 (“Applied to me very much or most of the time”) based on the past week. Higher scores indicate greater depressive symptoms. The reliability of the scale in Jun et al.'s study was Cronbach's *α* = 0.87, and in this study, it was 0.84.

### 5.3 Perceived Stigma

Perceived stigma was measured using the Korean version of the Infertility Stigma Scale (K‐ISS), developed by Fu et al. (Fu et al. 2015) and validated by Kim and Ban (Kim and Ban 2022). This tool includes 25 items across four subscales: self‐devaluation, public stigma, social withdrawal, and family stigma. Each item is rated on a 5‐point Likert scale from 1 (“Strongly disagree”) to 5 (“Strongly agree”), with higher scores indicating greater perceived stigma. The Cronbach's *α* of the Korean version was 0.97 at the time of validation (self‐devaluation = 0.86, social withdrawal = 0.77, public stigma = 0.92, family stigma = 0.84), and 0.96 in this study (self‐devaluation = 0.91, social withdrawal = 0.86, public stigma = 0.94, family stigma = 0.80).

### 5.4 Spousal Support

Spousal support perceived by participants was measured using the scale developed by Nam (Nam 1988) and revised by Park (Park 2007) for women undergoing infertility treatment. The scale consists of 23 items: 9 items on emotional support (love, respect, and trust), 2 items on support through sufficient communication, and 12 items on tangible support and care. Each item is rated on a 5‐point Likert scale from 1 (“Strongly disagree”) to 5 (“Strongly agree”), with higher scores indicating higher levels of spousal support. Cronbach's *α* was 0.95 in Park's study and 0.94 in this study.

## 6 Data Analysis

Data were analyzed using SPSS version 26.0 (IBM Corp., Armonk, NY, USA). Hayes' PROCESS macro (version 4.3) (Hayes 2022) was used to examine mediation and moderated mediation effects.

Specific analysis procedures were as follows:

Descriptive statistics including frequency, percentage, mean, and standard deviation were used to summarize participants' general characteristics and key variables (uncertainty, perceived stigma, spousal support, and depression).

Differences in key variables according to general and infertility‐related characteristics were analyzed using independent *t*‐tests and one‐way ANOVA.

Pearson's correlation coefficients were used to examine relationships among major variables.

PROCESS macro models 4 and 7 were used to test the mediation effect of perceived stigma in the relationship between uncertainty and depression, and the moderated mediation effect of spousal support.

The statistical significance of mediation effects was evaluated using bootstrapping with 95% confidence intervals (CIs).

## 7 Ethical Considerations

This study was approved by the Institutional Review Board (IRB) of Dankook University (DKU 2021‐10‐024). Participation in the study was voluntary. After providing a full explanation of the study's purpose, procedures, the right to withdraw at any time, and assurances of confidentiality and anonymity, written informed consent was obtained. Participants were informed they could withdraw at any time without any disadvantage. Surveys were conducted in a quiet, private setting to ensure participants' comfort, and small tokens of appreciation were provided. All collected data were anonymized and securely stored for research purposes only. This study complied with ethical guidelines, including the Declaration of Helsinki.

## 8 Results

### 8.1 Differences in Uncertainty, Depression, Stigma, and Spousal Support According to General and Infertility‐Related Characteristics

Among the participants, 74.6% were aged 35 years or older, and 52.5% had been married for less than five years. Employed participants accounted for 67.8%, while 46.6% reported having a religion. Regarding infertility‐related characteristics, 33.9% had undergone IVF two times or less, 40.7% had undergone IVF three to six times, and 25.4% had undergone IVF seven times or more. The most common cause of infertility was unexplained infertility (44.1%). Government financial support for infertility treatment was received by 61.0% of participants, and 33.1% had experienced miscarriage after infertility treatment. Only 9.3% had received counseling for infertility. Concerning the financial burden of infertility treatment, 31.4% responded “very burdensome,” while 44.1% responded “somewhat burdensome.” Regarding spousal involvement, 74.6% perceived their spouse as active, and 6.8% perceived them as passive.

Analysis of differences in dependent variables based on participants' general and infertility‐related characteristics revealed that depression significantly differed according to religious affiliation (*t* = 2.38, *p* = 0.019), experience of miscarriage (*t* = −2.05, *p* = 0.043), and receipt of government support for infertility treatment (*t* = −2.15, *p* = 0.036) (Table 1).

**TABLE 1:** The scores of uncertainty, depression, perceived stigma, spousal support according to general characteristics (*N* = 118).

Characteristics		*n* (%)	Uncertainty		Depression		Perceived stigma		Spousal support	
			Mean ± SD	t/F (*p*)	Mean ± SD	t/F (*p*)	Mean ± SD	t/F (*p*)	Mean ± SD	t/F (*p*)
Age	< 35 years	30 (25.4)	2.24 ± 6.25	−1.16 (0.248)	1.55 ± 1.22	0.17 (0.862)	1.77 ± 0.60	−0.83 (0.408)	4.07 ± 0.62	0.49 (0.628)
	≥ 35 years	88 (74.6)	2.39 ± 5.96		1.47 ± 1.18		1.90 ± 0.76		4.01 ± 0.57	
Duration of marriage	≤ 5 years	56 (47.5)	2.32 ± 0.60	−0.58 (0.563)	1.59 ± 1.10	0.85 (0.397)	1.90 ± 0.68	0.47 (0.639)	4.05 ± 0.54	0.47 (0.638)
	> 5 years	62 (52.5)	2.38 ± 0.61		1.41 ± 1.26		1.85 ± 0.77		4.00 ± 0.62	
Job	No	38 (32.2)	2.38 ± 0.50	0.42 (0.672)	1.51 ± 1.18	−0.09 (0.926)	1.96 ± 0.78	0.93 (0.354)	3.94 ± 0.59	−1.05 (0.298)
	Yes	80 (67.8)	2.33 ± 0.65		1.49 ± 1.20		1.83 ± 0.70		4.07 ± 0.58	
Religion	No	63 (53.4)	2.39 ± 0.66	0.84 (0.401)	1.73 ± 1.21	2.38 (0.019)	1.98 ± 0.79	1.86 (0.066)	4.03 ± 0.61	0.11 (0.913)
	Yes	55 (46.6)	2.30 ± 0.53		1.22 ± 1.11		1.74 ± 0.63		4.02 ± 0.55	
Number of IVF treatment	2^a^	40 (33.9)	2.15 ± 0.58	4.02 (0.021)	1.23 ± 1.00	1.94 (0.149)	1.60 ± 0.60	4.60 (0.012)	4.13 ± 0.52	1.03 (0.361)
	3 ~ 6^b^	48 (40.7)	2.40 ± 0.62	a < c	1.54 ± 1.21		1.99 ± 0.67	a < b, c	3.98 ± 0.60	
	≥ 7^c^	30 (25.4)	2.54 ± 0.54		1.78 ± 1.33		2.05 ± 0.87		3.96 ± 0.63	
Infertile factor	Unexplained	52 (44.1)	2.32 ± 0.65	0.56 (0.644)	1.34 ± 1.12	1.13 (0.341)	1.79 ± 0.64	1.04 (0.376)	3.90 ± 0.58	2.21 (0.091)
	Female factor	23 (19.5)	2.46 ± 0.53		1.86 ± 1.05		2.05 ± 0.67		4.23 ± 0.47	
	Male factor	5 (4.2)	2.10 ± 0.52		1.26 ± 1.21		1.54 ± 0.52		4.28 ± 0.30	
	Mixed factor	38 (32.2)	2.36 ± 0.59		1.52 ± 1.34		1.91 ± 0.87		4.05 ± 0.64	
Beneficiary of government	No	46 (39.0)	2.20 ± 0.52	−2.20 (0.030)	1.22 ± 1.20	−2.05 (0.043)	1.78 ± 0.76	−1.13 (0.259)	3.93 ± 0.68	−1.42 (0.159)
Subsidy	Yes	72 (61.0)	2.45 ± 0.63		1.67 ± 1.15		1.93 ± 0.70		4.09 ± 0.51	
Miscarriage experience after fertility treatment	No	79 (66.9)	2.30 ± 0.56	−1.39 (0.168)	1.32 ± 1.11	−2.15 (0.036)	1.83 ± 0.72	−0.89 (0.376)	4.03 ± 0.53	0.07 (0.945)
	Yes	39 (33.1)	2.46 ± 0.67		1.84 ± 1.28		1.96 ± 0.74		4.02 ± 0.69	
Experience of counseling	No	107 (90.7)	2.33 ± 0.61	−0.92 (0.359)	1.43 ± 1.16	−1.81 (0.073)	1.82 ± 0.68	−2.46 (0.015)	4.06 ± 0.52	0.95 (0.362)
For infertility	Yes	11 (9.3)	2.51 ± 0.47		2.10 ± 1.31		2.37 ± 0.94		3.75 ± 1.03	
Treatment cost affordability	Easily^a^	29 (24.6)	2.33 ± 0.50	0.95 (0.389)	1.25 ± 1.18	1.90 (0.154)	1.71 ± 0.65	1.48 (0.234)	3.92 ± 0.57	3.69 (0.028)
	Medium^b^	52 (44.1)	2.28 ± 0.64		1.42 ± 1.15		1.86 ± 0.73		4.19 ± 0.47	b > a, c
	Hardly^c^	37 (31.4)	2.46 ± 0.63		1.79 ± 1.22		2.02 ± 0.77		3.88 ± 0.69	

*Note:* Number of IVF treatment: a, twice; b, 3 to 6 times; c, 7 or more times Note for Treatment cost affordability: a, Easily; b, Medium; c, Hardly.

### 8.2 Correlations Among Uncertainty, Depression, Stigma, and Spousal Support

Correlation analysis showed that depression was positively correlated with stigma (*r* = 0.58, *p* < 0.001) and uncertainty (*r* = 0.53, *p* < 0.001), and negatively correlated with spousal support (*r* = −0.26, *p* = 0.005). The mean scores for the key variables were: depression = 2.35 ± 0.60, stigma = 1.49 ± 1.19, uncertainty = 1.87 ± 0.73, and spousal support = 4.03 ± 0.58 (Table 2).

**TABLE 2:** Correlation and descriptive statistics of the research variables (*N* = 118).

Variables
	*r* (*p*)				Range of scale	M ± SD
Uncertainty	Depression	Perceived Stigma	Spousalsupport			
Uncertainty	1				1 ~ 5	2.35 ± 0.60
Depression	0.530 (< 0.001)	1			0 ~ 3	1.49 ± 1.19
Perceived stigma	0.485 (< 0.001)	0.575 (< 0.001)	1		1 ~ 5	1.87 ± 0.73
Spousal support	−0.395 (< 0.001)	−0.256 (0.005)	−0.317 (< 0.001)	1	1 ~ 5	4.03 ± 0.58

### 8.3 Mediating Effect of Stigma in the Relationship Between Uncertainty and Depression

To analyze the mediating effect of stigma on the relationship between uncertainty and depression while controlling for miscarriage experience, counseling experience, and perceived spousal involvement (which showed significant differences in depression), PROCESS Macro Model 4 was employed. In Step I, uncertainty (independent variable, X) had a significant effect on depression (dependent variable, Y) (*β* = 0.93, *p* < 0.001). In Step II, uncertainty significantly affected stigma (mediator, M) (*β* = 0.56, *p* < 0.001). In Step III, stigma significantly affected depression even after controlling for uncertainty (*β* = 0.62, *p* < 0.001), indicating a significant indirect effect (mediating effect) of stigma in the relationship between uncertainty and depression (*β* = 3.83, *p* < 0.001). Bootstrapping with 10 000 resamples was conducted to verify the statistical significance of the mediating effect. The indirect effect coefficient was 0.35, and the 95% confidence interval (CI) ranged from 0.18 to 0.56, which did not include zero, indicating that the mediating effect of stigma was statistically significant (Table 3).

**TABLE 3:** Mediating effect of perceived stigma in the between uncertainty and depression (*N* = 118).

Model	Independent variable	Dependent variable	*β*	SE	*t*	*p*	R^2^	F (*p*)
Mediating effect								
Step I	X	Y	0.93	0.15	5.98	< 0.001	0.35	15.40 (< 0.001)
Step II	X	M	0.56	0.10	5.51	< 0.001	0.25	9.65 (< 0.001)
Step III	X, Y	Y					0.46	19.20 (< 0.001)
	X	Y	0.58	0.16	3.61	< 0.001		
	M	Y	0.62	0.13	4.76	< 0.001		
		Effect		Boot. SE		Boot. 95% CI(LLCI~ULCI)		
Indirect effect (X → M → Y)		0.35		0.10		0.18 ~ 0.56		

*Note:* CI, confidence interval; M, perceived stigma; SE, standard error; X, uncertainty; Y, depression.

### 8.4 Moderated Mediation Effect of Spousal Support in the Relationship Between Uncertainty and Depression via Stigma

A moderated mediation model, which integrates both mediation and moderation, was used to assess whether the indirect effect varied by the level of the moderator—in this case, spousal support. After controlling for miscarriage experience, counseling experience, and spousal involvement, PROCESS Macro Model 14 was used to examine the moderated mediation effect of spousal support on the relationship between uncertainty and depression via stigma.

Bootstrapping was used to determine the statistical significance of the conditional indirect effect and moderation effect, and the 95% CI of the moderated mediation index was calculated.

The results showed that in Step I, uncertainty (X) significantly influenced stigma (M) (*β* = 0.56, *p* < 0.001). The direct effect of uncertainty (X) on depression (Y) was significant (*β* = 0.54, *p* = 0.002), as was the effect of stigma (M) on depression (Y) (*β* = 0.61, *p* < 0.001). However, the direct effect of spousal support (W) on depression (Y) (*β* = −0.09, *p* = 0.617) and the interaction term (M × W) on depression (Y) (*β* = 0.02, *p* = 0.913) were not statistically significant.

The conditional indirect effects by spousal support level were as follows: Low spousal support (mean—1SD): effect = 0.34, 95% CI = [0.12, 0.54], Average spousal support: effect = 0.34, 95% CI = [0.17, 0.55], High spousal support (mean + 1SD): effect = 0.35, 95% CI = [0.15, 0.59].

**FIGURE 2:**
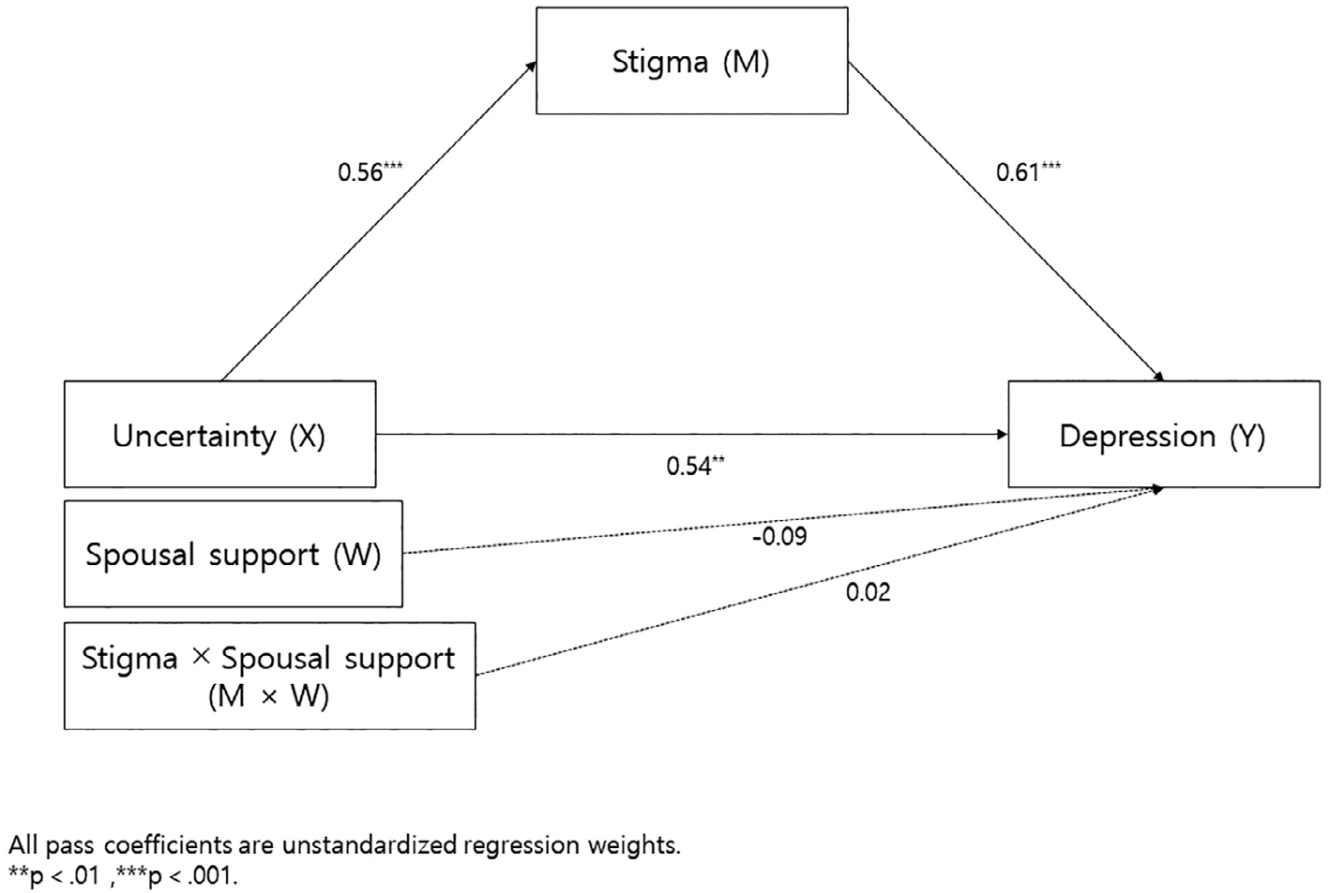
Mediating effect of perceived stigma between uncertainty and depression and moderated mediation effect of spousal support.

Although the indirect effects were statistically significant across all levels of spousal support, the moderated mediation index was 0.01 with a 95% CI of [−0.15, 0.21], which included zero. Therefore, the moderated mediation effect of spousal support was not statistically significant (Table 4, Figure 2).

**TABLE 4:** Moderated mediation effect analysis of spousal support (*N* = 118).

Model	Independent variable	Dependent variable	*β*	SE	*t*	*p*	R^2^	F (*p*)
Moderated mediation effect								
Step I	X	M	0.56	0.10	5.51	< 0.001	0.25	9.65 (< 0.001)
Step II	X	Y	0.54	0.17	3.15	0.002	0.46	13.54 (< 0.001)
	M	Y	0.61	0.13	4.55	< 0.001		
	W	Y	−0.09	0.19	−0.50	0.617		
	X × W	Y	0.02	0.16	0.11	0.913		
		Spousal support	Effect		Boot. SE	Boot. 95% CI (LLCI~ULCI)		
Conditional indirect effect		Mean–1SD (3.44)	0.34		0.11	0.12 ~ 0.54		
		Mean (4.03)	0.34		0.10	0.17 ~ 0.55		
		Mean + 1SD (4.61)	0.35		0.11	0.15 ~ 0.59		
Moderated mediation index = 0.0100							−0.15 ~ 0.21	

Abbreviations: Boot, bootstrapping; CI, confidence interval; LLCI, lower limit confidence interval; M, perceived stigma; SE, standard error; ULCI, upper limit confidence interval; W, spousal support; X, uncertainty; Y, depression.

## 9 Discussion

In this study, the level of uncertainty perceived by infertile women undergoing ART was similar to that reported in a previous study (Lee et al. 2020). Uncertainty was significantly higher among women who had undergone IVF more than seven times compared to those undergoing their second IVF attempt. This is consistent with prior research (Rooney and Domar 2016), which found that failed ART experiences increase uncertainty regarding future procedures and the likelihood of pregnancy success. These findings indicate that women consistently experience high levels of uncertainty throughout the ART process and that repeated treatment failures may diminish their confidence in achieving pregnancy. Therefore, active involvement from healthcare professionals is essential to alleviate emotional uncertainty across the ART process.

The participants in this study had all experienced at least one failed ART treatment cycle. Their levels of depression were higher than those reported in earlier studies that included women undergoing their first IVF attempt (Kim et al. 2024). This supports findings from a previous study (Sharma and Shrivastava 2022), which indicated that financial strain and mental health challenges worsen after the first IVF failure and during consideration of subsequent procedures. These results suggest that repeated treatment failures can increase psychological burdens in infertile women, highlighting the importance of ongoing emotional support and counseling throughout the ART process. Thus, nurses should assess women's emotional states at each treatment stage and provide more systematic and personalized psychological interventions, especially after treatment failures.

In this study, the perceived stigma among women undergoing ART was slightly lower than the midpoint and similar to the levels observed during the development of the Korean version of the stigma scale (Kim and Ban 2022). Moreover, perceived uncertainty, depression, and stigma showed positive correlations. This finding aligns with a study on women with primary ovarian insufficiency, where uncertainty was positively correlated with both stigma and depression (Davis et al. 2010). Furthermore, prior research has shown that how women perceive, interpret, and respond to uncertainty about infertility and its treatment can influence depression (Gentes and Ruscio 2011), and that perceived stigma is significantly related to depression (Ozturk et al. 2021).

In contrast, perceived spousal support showed negative correlations with levels of uncertainty, depression, and stigma. This supports prior findings indicating that spousal support has a positive impact on women's emotional difficulties (Patel et al. 2018; Nasim et al. 2019). Social expectations toward couples experiencing infertility, along with negative attitudes from family, friends, or society, or the pressure to pursue socially acceptable treatments, can threaten psychological well‐being. In such contexts, active spousal support can help reduce social pressure and contribute to emotional well‐being (Nasim et al. 2019). Therefore, mutual spousal support is crucial throughout the infertility diagnosis and treatment process. It is necessary to explore various supportive and educational interventions that help couples understand this need and actively cope and overcome challenges (Kucukkelepce and Unver 2022). Although spousal support was conceptualized as a moderating variable in this study, it may also influence the level of uncertainty itself. Supportive spousal relationships may help alleviate infertility‐related uncertainty throughout the ART process through shared understanding, emotional reassurance, and collaborative decision‐making. From this perspective, spousal support may function not only as a buffering factor that mitigates psychological distress but also as an antecedent that shapes women's perceptions of uncertainty. Future studies should employ diverse analytical models, such as longitudinal research designs, to better capture the dynamic role of spousal support in infertility‐related psychological processes.

This study also found that perceived stigma mediated the relationship between uncertainty and depression in women undergoing ART. This is consistent with previous research indicating that perceived stigma is a significant predictor of depression among infertile women (Rooney and Domar 2016; Yokota et al. 2022). Infertile women face psychological challenges due to various factors such as uncertainty regarding the cause of infertility, the duration of treatment, and financial stress (Patel et al. 2018). In such situations, infertility‐related stigma can lead to psychological distress, including anxiety and depression, as well as reduced quality of life and social isolation, thereby negatively affecting individual health (Ozturk et al. 2021; Lin et al. 2022). In other words, various uncertainties stemming from infertility reinforce internal and external perceptions of stigma, which ultimately lead to depression.

These uncertainties, resulting from infertility, foster perceived stigma both from within and from society, causing women to experience depression. In many cultures, being childless is considered an undesirable social role, and infertility is viewed as an “unexpected life transition” (Koropatnick et al. 1993). Infertility, as a stigmatized condition, leads women to internalize negative beliefs about themselves, resulting in depression, lowered self‐esteem, and decreased quality of life. In severe cases, this may lead women to delay or discontinue infertility treatments and give up on pregnancy (Hwang 2013). Especially in dominant cultures, perceptions of being devalued by others or cues suggesting such devaluation threaten one's sense of identity. When women are unable to cope effectively, these threats may result in anxiety and depression (Major and O'Brien 2005). Previous studies have emphasized the need for healthcare providers to deliver psychoeducational interventions to reduce self‐stigma in women receiving infertility treatment. Additionally, public anti‐stigma campaigns are necessary to reduce social stigma toward infertile women (Yokota et al. 2022). Therefore, psychological interventions, including early detection of emotional difficulties and cognitive therapy, should be recognized as essential components—not optional—in the management of infertility.

Finally, this study analyzed whether the indirect effect of uncertainty on depression through perceived stigma varied depending on the level of spousal support. The results showed that the indirect effect was statistically significant regardless of the spousal support level (low, average, high), but there was no significant difference in the magnitude of the indirect effect across these levels. Moreover, the 95% confidence interval for the index of moderated mediation included zero, indicating that the moderated mediation effect was not statistically significant. This suggests that the strength of the indirect pathway from uncertainty to depression via stigma did not differ meaningfully according to the degree of spousal support.

This study has several limitations. First, due to the cross‐sectional design of this study, causal relationships among uncertainty, perceived stigma, spousal support, and depression cannot be established. Future longitudinal studies are needed to clarify the directionality and dynamic interplay among these psychological factors across different stages of infertility treatment. Second, as participants were recruited from a single fertility clinic in one region, caution is warranted in interpreting and generalizing the findings. Women undergoing infertility treatment may experience different levels of uncertainty, perceived stigma, and spousal support depending on regional, healthcare system, and cultural contexts. Although spousal support was modeled as a moderating variable in this study, it may also have a direct influence on the formation of perceived uncertainty. This should be considered a limitation in interpreting the proposed research model.

## 10 Conclusion

This study aimed to examine the mediating effect of perceived stigma in the relationship between uncertainty and depression among women undergoing ART, as well as the moderated mediation effect of spousal support. Through this study, it was found that perceived stigma plays a mediating role between uncertainty and depression; however, spousal support does not exert a moderated mediation effect on the strength of this mediation. Therefore, in order to alleviate the psychological distress of women undergoing infertility treatment, it is essential to first minimize uncertainty related to the diagnosis, treatment options, and prognosis of infertility. Furthermore, strategies should be prioritized that help women restore their inner sense of identity and adopt active coping mechanisms by positively accepting infertility, thereby reducing perceived stigma. These approaches are expected to contribute to the strength and sustainability of positive psychological outcomes throughout the course of infertility treatment.

Future studies with larger sample sizes are recommended. In addition, since stigma related to infertility may vary depending on social norms, expectations, and cultural contexts, and the nature of stigma can be influenced by various temporal, situational, and social factors—such as the stage of treatment, cause of infertility, duration of infertility, number of treatment failures, and timing of the survey—it is suggested that future research take these elements into comprehensive consideration.

## 11 Relevance for Clinical Practice

Depression in women undergoing assisted reproductive technology is influenced by uncertainty and perceived stigma, while spousal support may not always buffer these effects. Clinical interventions should focus on early identification of psychological distress, reducing stigma, and addressing uncertainty. Multidimensional strategies to enhance not only emotional but also practical spousal support are essential.

## Author Contributions

**Miok Kim:** conceptualization, funding acquisition, writing – original draft, investigation, methodology, validation, visualization, writing – review and editing, project administration, formal analysis, data curation, supervision.

## Funding

The present research was supported by the research fund of Dankook University in 2025 (No. R202500697).

## Ethics Statement

The study was approved by the Dankook University in Korea, approval number [DKU 2021‐10‐024].

## Consent

Informed consent was obtained from all individual participants included in the study.

## Conflicts of Interest

The author declares no conflicts of interest.

## Data Availability

The data that support the findings of this study are available on request from the corresponding author. The data are not publicly available due to privacy or ethical restrictions.
